# American Illustrated Medical Dictionary

**Published:** 1915-10

**Authors:** 


### BIBLIOGRAPHY

AMERICAN ILLUSTRATED MEDICAL DICTIONARY. This is the eighth revision of this popular medical dictionary. It shows considerable revision and many additional terms, most of which, however, are for the most part to render the book more easily used and to present in tabular form condensed information. There is one feature of some dental interest that we can not so cordially indorse, and that is the inclusion of definitions of proprietary remedies. There is no good reason why pyrozone, pyorrocide, listerine, lysol and other such proprietary preparations should be defined in a general dictionary of medical scientific terms. Because of the fact that these remedies are often prescribed by medical and dental practitioners hardly warrants their inclusion in a dictionary that is to be used largely by medical and dental students. This criticism, however, may be made of all medical dictionaries. The students' edition is also revised and makes a convenient and handy reference book, for busy practitioners although the definitions are almost too brief to convey the full meaning of unusual terms. Both books are well made and are published by the well known W. B. Saunders Co., of Philadelphia.

